# Genus *Echinophora*—Biological Activity, Chemical Composition, and Future Perspectives

**DOI:** 10.3390/plants13121599

**Published:** 2024-06-08

**Authors:** Stanislava Ivanova, Stanislav Dyankov, Rayna Ardasheva, Kalin Ivanov

**Affiliations:** 1 Department of Pharmacognosy and Pharmaceutical Chemistry, Faculty of Pharmacy, Medical University-Plovdiv, 4002 Plovdiv, Bulgaria; stanislav.dyankov@mu-plovdiv.bg (S.D.); kalin.ivanov@mu-plovdiv.bg (K.I.); 2 Research Institute, Medical University-Plovdiv, 4002 Plovdiv, Bulgaria; 3 Department of Medical Physics and Biophysics, Faculty of Pharmacy, Medical University-Plovdiv, 4002 Plovdiv, Bulgaria; rayna.ardasheva@mu-plovdiv.bg

**Keywords:** *Echinophora*, traditional medicine, novel drugs, phytopharmaceuticals

## Abstract

Species belonging to the genus *Echinophora* (Apiaceae) have been used by humanity since ancient times as flavoring agents, preservatives, and medicines for the treatment of a broad spectrum of conditions, including respiratory, digestive and kidney disorders, fungi infections, wounds, and gastric ulcers. Phytochemical studies on this botanical genus mainly investigate the essential oil composition and reveal the species as a rich source of volatile compounds, including monoterpenes and phenylpropanoids. Among the non-volatile secondary metabolites, flavonoids, coumarins, phenolic acids, phytosterols, and polyacetylenes have been identified. Pharmacological studies revealed *Echinophora* extracts and essential oils exhibit different biological properties, including antibacterial, antifungal, anticancer, insecticidal, anti-inflammatory, and hepatoprotective activities. However, compared to other genera, the biological activity and chemical diversity of this genus are not well studied. In future decades, it is highly likely that the small genus *Echinophora* will play an important role in drug discovery and drug development.

## 1. Introduction

The genus *Echinophora* belongs to the Apiaceae (Umbelliferae) family and consists of a small number of plant species, including *Echinophora platyloba* DC., *Echinophora cinerea* (Boiss.) Hedge & Lamond, *Echinophora chrysantha* Freyn & Sint., *Echinophora scabra* Gilli, *Echinophora spinosa* L., *Echinophora tenuifolia* L., *Echinophora lamondiana* Yildiz & Z. Bahcecioglu, *Echinophora trichophylla* Sm., *Echinophora tournefortii* Jaub. & Spach, *Echinophora tenuifolia* subsp. *sibthorpiana* (Guss.) Tutin or *Echinophora sibthorpiana* Guss., and *Echinophora orientalis* Hedege & Lamond [1].

**Figure 1:**
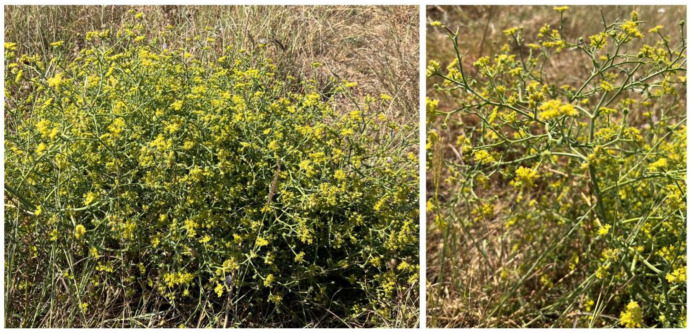
*Echinophora tenuifolia* subsp. *sibthorpiana* (Guss.) Tutin.

The name *Echinophora* originates from ancient Greek and is composed of the words echino (spine) and phora (leaf), which describe the spiny leaves of the species within the genus [2]. The genus is distributed in regions of the Mediterranean and the Balkan Peninsula, Anatolia, eastward to Iran and Afghanistan [3]. Three of the species, namely *E. chrysantha*, *E. lamondiana*, and *E. trichophylla*, are endemic to Turkey, while *E. platyloba* and *E. cinerea* are endemic to Iran [1,3]. In Europe, the genus is represented by *E. spinosa*, *E. tenuifolia*, and *E. tenuifolia* subsp. *sibthorpiana* (or *E. sibthorpiana*) (Figure 1), the latter being the most widely distributed species from the genus that can be found in numerous countries, including countries of the Balkans (Greece, Bulgaria, and North Macedonia), Turkey, and Iran [1,3,4,5,6]. *E. spinosa* is a halophytic plant distributed in the sandy coastal regions of the Mediterranean, found in South Europe (including France, Italy, Montenegro, and Greece), as well as the coastal regions of Northern Africa, including Algeria, and more recently, Tunisia [7,8,9,10].

Since ancient times, *Echinophora* species have been used by humanity as flavoring agents, preservatives, and medicines [1]. Compared to other genera, the biological activity and chemical diversity of *Echinophora* are not well studied. Currently, plants belonging to this genus are considered promising candidates for novel phytopharmaceuticals. Some of the target directions of *Echinophora* species are oncology [11,12,13], cardiology [14], gynecology [15], and gastroenterology [16]. Previous studies established that *Echinophora* extracts contain molecules called echinophorins [2,17]. It was found that these compounds demonstrate significant selectivity toward the transient receptor potential channels of ankyrin type-1 (TRPA1). Echinophorins are considered to have a promising potential in the management of pain and inflammation [2].

The aim of this study is to review the phytochemistry, biological activity, and pharmacologic properties of *Echinophora* species.

## 2. Results and Discussion

### 2.1. Chemical Diversity of the Metabolites Produced by Plants from Echinophora Genus

#### 2.1.1. Secondary Metabolites in *Echinophora* Extracts

The Apiaceae family is characterized by the production of distinctive secondary metabolites such as coumarins and furanocoumarins, polyacetylenes, volatile phenylpropenes, etc. [18]. Phytochemical studies regarding the composition of *Echinophora* extracts are limited. In general, the main compounds identified in extracts of this botanical genus are polyphenolic compounds, including phenolic acids, flavonoids, and coumarins, as well as polyacetylenes, fatty acids, and terpenoids, namely phytosterols [2,11,19,20,21,22,23,24,25]. An overview of the secondary metabolites identified and isolated from extracts of *Echinophora* species is depicted in Table 1.

Several flavonoids, including quercitrin, rutin, hesperidin, quercetin-3-*O*-glucoside, quercetrin-3-*O*-β-D-glucopyranoside, and kaempferol-3-*O*-β-D-glucopyranoside, were identified in extracts from *E. tenuifolia* [11,19], *E. cinerea* [20,21], *E. platyloba* [23], *E. chrysantha* [24], and *E. tournefortii* [25]. Flavonoids are widely distributed hydroxylated plant secondary metabolites that possess various biological activities and health benefits [26]. The flavonol glycosides quercitrin (or quercetin-3-*O*-rhamnoside) and rutin (or quercetin-3-*O*-rutinoside) have been identified and quantified in polar ethyl acetate fraction of methanolic extract from inflorescences of *E. tenuifolia* [19]. In addition, rutin was present in the dichloromethane fraction of methanolic extract from the branches of the same plant [11]. Rutin was also identified as the most abundant flavonoid in hydroalcoholic extract from *E. chrysantha*, in addition to quercetin-3-*O*-glucoside and the flavanone glycoside hesperidin being present in significant quantities [24]. The aglycone quercetin was found in samples from *E. platyloba* prepared by hollow fibre-based liquid phase microextraction after hydrolysis with HCl [23]. The flavonol glucopyranosides quercetrin-3-*O*-β-D-glucopyranoside and kaempferol-3-*O*-β-D-glucopyranoside were isolated from the hydroalcoholic extract of *E. cinerea* [20,21]. In addition to flavonoids, the *E. cinerea* extract contained the coumarin osthol and the furanocoumarin isoimperatorin [19,20]. Phenolic acids were described in *Echinophora* extracts, including ferulic acid from *E. tenuifolia* [19] and 2,5-dihydroxybenzoic and caffeic acid in *E. tournefortii* [25]. Eleven phenolic acids were identified in *E. chrysantha*, with quinic acid and chlorogenic acid being the most abundant [24].

**Figure 2:**
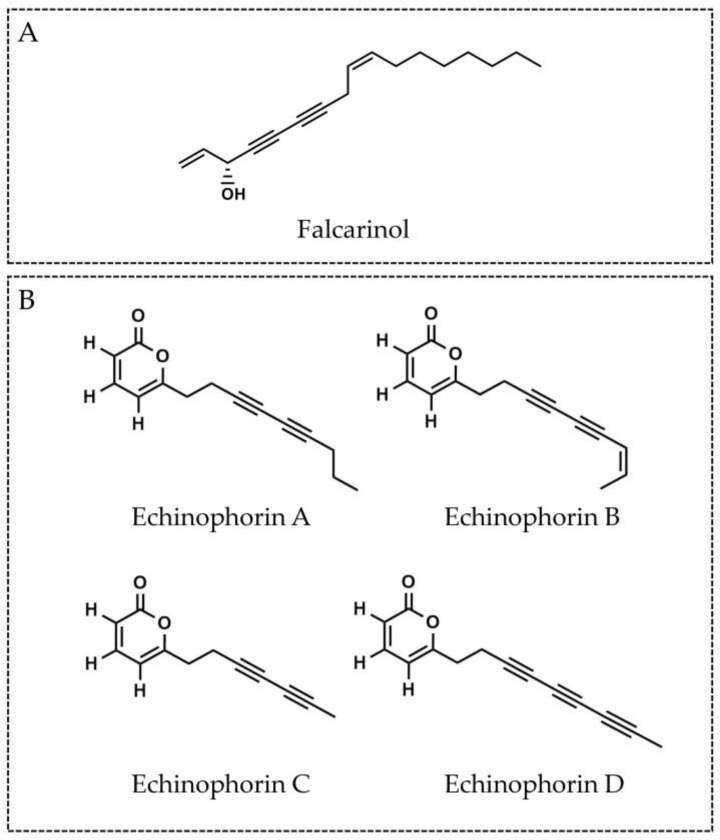
Structures of polyacetylenes from the *Echinophora* genus (**A**) Falcarinol and (**B**) Echinophorins A–D, isolated from *E. cinerea* and *E. platyloba* [2,17].

Characteristic bioactive chemical compounds for the Apiaceae family are polyacetylenes, mainly represented by the falcarinol-type C_17_-polyacetylenes [27]. The archetypal C_17_-polyacetylene falcarinol is an aliphatic alcohol biosynthesized from fatty acids, the structure of which contains two triple bonds and two double bonds (Figure 2A) [2,27]. A less common class of polyacetylenes, the C_14_-polyacetylenes, have been described in extracts of different plant species, including *Codonopsis pilosula* [28], *Coreopsis tinctoria* [29,30], and species from the genus *Lobelia* and the genus *Siphocampylus* [31]. Unique among the C_14_-polyacetylenes are the C_14_-polyacetylenes containing α-pyrone moiety, first described in *Mediasia macrophylla* [32] and consequently found in the Iranian *Echinophora* species *E. cinerea* and *E. platyloba* [2,17,20]. The echinophorins echinophorin A, echinophorin B, and echinophorin C, a type of α-pyrone containing C_14_-polyacetylenes named after the genus, were first isolated from acetone extract of *E. cinerea* [17]. Echinophorin A and echinophorin B were later isolated from the dichloromethane fraction of *E. platyloba* extract in addition to one new echinophorin—echinophorin D [2]. To date, no other echinophorins have been described. Similar to other polyacetylenes like falcarinol, the structure of echinophorins includes two or three conjugated triple bonds, but it differs by the presence of an α-pyrone ring, which is a unique feature among this class of compounds [2]. The structures of the echinophorins, isolated from *E. cinerea* and *E. platyloba*, are presented in Figure 2B. Besides echinophorines, the C_17_-polyacetylene falcarinol was identified as one of the main constituents of the essential oil isolated from the roots of *E. spinosa*, as well as in essential oil from the roots of *E. orientalis* [7,33,34,35]. 

#### 2.1.2. Volatile Constituents of *Echinophora* Essential Oils

One of the main features of the large Apiaceae family, to which the genus *Echinophora* belongs, is the production of volatile terpenoids, and many of the species within this family are excellent sources of essential oils (EOs) [36]. Most of the phytochemical studies of the species within this genus are focused on the composition of their EOs. The major volatile constituents identified in the isolated EOs from *Echinophora* species are summarized in Table 2. 

The main compounds found in the EO of *E. tenuifolia* subsp. *sibthorpiana* are methyl eugenol, δ-3-carene, α-phellandrene, as well as *p*-cymen, with the differences in their content in EOs from different geographical regions being mainly quantitative [4,5,6,37,38,39,40,41,42,43,44,45]. The phenylpropanoid methyl eugenol was found to be the major constituent in EOs from Bulgaria [5], Iran [37], Republic of North Macedonia [6], and Turkey [41]. The monoterpene α-phellandrene was the main constituent in the EOs from plants found in Greece [4] and Turkey [40], while δ-3-carene was found in significant quantity in EO from Turkey [40]. A unique EO profile was found in *E. tenuifolia* subsp. *sibthorpiana* population from Crete Island, Greece, in which the main constituents of the EO were β-phellandrene and α-pinene, while germacrene D and bornyl acetate were reported for the first time as a major constituents in the EO [46].

The chemical composition of *E. tenuifolia* subsp. *sibthorpiana* EO varies depending on different factors such as the vegetative stage of the plant, the drying conditions, the harvest location, the year of collection of the plant, and storage conditions [38,40,42,43]. Şanlı et al. compared the composition of the EO changes depending on the growth stage of the plant (rosette, vegetative growth, flowering, or fruit-ripening) and the drying conditions (fresh plant, shade-dried, and sun-dried) and found that the content of methyl eugenol decreased from the rosette stage to the fruit-ripening stage, being the highest in the rosette stage, while the content of α-phellandrene increased, being the highest at the flowering stage [38]. Moreover, the content of methyl eugenol was the highest in the EO isolated from fresh plants and decreased during drying (shade and sun drying) in all flowering stages, whereas the content of α-phellandrene increased significantly during drying. Özcan and Akgül compared the chemical composition of the EOs from plants collected in different months (April, May, and June)—the quantity of both methyl eugenol and α-phellandrene in the EOs increased during the period of the study, being the highest in June (25.96% and 29.96%, respectively) [40]. Additionally, the yield and composition of the EO are significantly affected by the cultivation of the plant [44]. The cultivated *E. tenuifolia* subsp. *sibthorpiana* showed higher EO yield in addition to changes in the EO composition—the content of the main constituent in the wild plant δ-3-carene decreased dramatically in the cultivated plant, while that of α-phellandrene increased [44].

Apart from methyl eugenol, four other members of the phenylpropanoid class of volatile compounds have been detected in considerable amounts in species of the *Echinophora* genus, specifically myristicin in *E. spinosa* and *E. orientalis* [7,8,35,47] and asarone, anethole, and eugenol in *E. platyloba* [48]. Myristicin, terpinolene, and the polyacetylene (*Z*)-falcarinol are the dominant compounds in the root EO of *E. spinosa* [7,33]. Myristicin was the principal compound in the root EO (47.4%) of *E. spinosa* growing in Corsica Island, France, while its content in the aerial parts EO was notably lower (2.7%) [7]. In addition to myristicin, the root EO contained significant amounts of terpinolene and (*Z*)-falcarinol and negligible amounts of *p*-cymene (0.3%), which was the most abundant compound of the aerial part EO (35.8%) along with α-phellandrene (21.7%) [7]. Notable differences between the composition of the root and aerial parts EOs from *E. spinosa* growing on the Island of Sicily were observed as well [33]. While the root EO was characterized by the predominant compounds myristicin, terpinolene, and (*Z*)-falcarinol, the major constituents of the aerial parts EO were α-phellandrene and *p*-cymene [33]. Interestingly, the EO from roots of *E. orientalis* from Iran showed a similar profile to the *E. spinosa* root EO, with myristicin, terpinolene, and falcarinol being the major compounds identified [35]. In the aerial parts and ripe fruit EOs of *E. spinosa* from central Italy, the content of myristicin was 16.5% and 15.3%, respectively [8]. The main constituent of the aerial parts EO was β-phellandrene (34.7%), while the greatest fraction of the fruit EO was represented by *p*-cymene (50.2%) [8]. The principal constituent of aerial parts *E. spinosa* EO from Montenegro was δ-3-carene (60.86%), whereas the content of myristicin was low (1.04%) [47].

The main volatile constituent detected in the EO of the endemic Iranian species *E. cinerea* is α-phellandrene, with α-pinene, *p*-cymene, and β-phellandrene being found in significant amounts [49,50,51,52,53]. In a comparative study of *E. cinerea* EOs isolated from plants in different growth stages (early flowering stage and full flowering stage) from different populations in Iran, it was found that the yield of the EO and the content of α-phellandrene was the highest during the full flowering stage of the plant [49]. In another study comparing the method of extraction of the EO, the content of α-phellandrene in the EO extracted via headspace solvent microextraction (HD–SME) was significantly higher than that extracted with conventional hydro-distillation [53].

The chemical composition of the EO from the other endemic Iranian species, *E. platyloba*, is more diverse [48,54,55,56,57]. Sodeifian and Sajadian compared the composition of EO from *E. platyloba* extracted using two different methods (supercritical carbon dioxide extraction and hydro-distillation) [54]. The major constituents in the EO extracted using supercritical carbon dioxide were linalool (16.02%), *trans*-β-ocimene (11.58%), α-pinene (7.10%), 2,4,6-trimethylanisole (6.98%), and spathulenol (5.29%). In comparison, the EO extracted by hydro-distillation contained significantly higher amounts of *trans*-β-ocimen (46.99%) and spathulenol (9.04%), while the major constituent of the EO extracted using supercritical carbon dioxide linalool was not detected [54]. Thymol was found to be the main constituent of *E. platyloba* EO from southwest Iran [56]. In contrast, a different EO profile of *E. platyloba* was described in a population from northeastern Iran, where the main constituents of the EO were the phenylpropanoids asarone (10.15%), anethole (7.39%), and eugenol (6.74%) [48]. In the EO isolated from *E. platyloba* seeds, the most abundant volatile compound was *p*-cymen (22.15%), followed by α-pinene, β-phellandrene, and α-phellandrene [58].

**Table 2:** Comparison of the main volatile compounds in EOs of *Echinophora* species.

Plant	Plant Part	Reported Yields	Main Phytochemicals	Other Compounds	Refs.
*E. tenuifolia* subsp. *sibthorpiana*	Aerial parts	0.43–2.09%	methyl eugenol, δ-3-carene, α-phellandrene	*p*-cymene, *o*-cymene, α-pinene, β-phellandrene	[4,5,6,37,38,40,41,42,44,45,46]
	Leaves	0.77%	δ-3-carene, methyl eugenol, α-phellandrene	*p*-cymene, α-phellandrene-8-ol	[39,43]
*E. cinerea*	Aerial parts	0.2–2.05%	α-phellandrene, α-pinene, β-phellandrene, β-myrcene	*p*-cymene, linalool, citronellol	[49,50,51,52,53]
	Stems	0.17%	α-phellandrene, β-phellandrene, *p*-cymene	α-pinene, linalool, β-pinene	[49]
	Leaves	0.21%	α-phellandrene, α-pinene, β-phellandrene	*p*-cymene, myrcene, β-pinene	
	Flowers	-	α-phellandrene, α-pinene, β-phellandrene	*p*-cymene, β-pinene, linalool	
*E. platyloba*	Aerial parts	0.3–1.12%	β-ocimene, thymol, α-pinene, limonene, asarone, *p*-cymene	linalool, δ-3-carene, anethole, eugenol, spathulenol	[48,54,55,56,57,59]
	Seeds	0.8%	*p*-cymene, α-pinene, β-phellandrene	α-phellandrene, carvacrol	[58]
*E. spinosa*	Aerial parts	0.2–0.83%	α-phellandrene, δ-3-carene, *p*-cymene, β-phellandrene	α-pinene, myristicin	[7,8,33,47]
	Roots	0.1%	myristicin, terpinolene, (*Z*)-falcarinol	-	[7,33]
	Ripe fruits	1.13%	*p*-cymene, α-pinene, α-phellandrene	β-myrcene	[8]
*E. chrysantha*	Aerial parts	0.65–1.5%	α-phellandrene, β-phellandrene	*p*-cymene	[60,61,62]
*E. tournefortii*	Aerial parts	0.18%	α-pinene, caryophyllene oxide, myrcene	*trans*-verbenol, β-pinene,	[61,63]
*E. lamondiana*	Aerial parts	0.54–2.09%	δ-3-carene, α-phellandrene	*p*-cymene, terpinolene	[64,65]
*E. orientalis*	Aerial parts	0.48%	myrcene, *p*-cymene	limonene, β-phellandrene	[61]
	Seeds	0.45%	spathulenol, carotol	bicyclogermacrene, germacrene D, α-humulene	[35]
	Shoots	1.0%	myrcene, *p*-cymene	3,6-dimethylene-1,7-octadiene, α-pinene, α-phellandrene	
	Roots	1.0%	myristicin, terpinolene	falcarinol, myrcene	

### 2.2. Ethnobotanical Importance and Ethnomedicinal Use

The species within the genus *Echinophora* have a long history of traditional use, including ethnomedicinal use, specifically *E. platyloba* in Iran [66] and *E. tenuifolia* subsp. *sibthorpiana* in Turkey [67]. The ethnomedicinal use of these plants is summarized in Table 3.

*E. platyloba*, also known as “Khosharizeh” or “Khosharouzeh” in Persian, is used in the cuisine of Iran as a spice, food flavoring agent in dairy products, and as a preservative in tomato paste and pickles [58,66,68]. Moreover, *E. platyloba* has been reported in various ethnobotanical and ethnomedicinal studies for its use as a medicinal plant in many different regions of Iran. It is used for relieving the symptoms of respiratory conditions like cough and common cold, digestive disorders like ileus, flatulence, diarrhea, and hemorrhoids, in the treatment of kidney stones and kidney pain, joint pain, mouth ulcers, and as an antifungal agent [68,69,70,71,72,73,74]. *E. cinerea*, also known as “Khosharizeh-e-Kohestani” in Persian, is used in the folk medicine of Iran as a stimulant and an invigorator of the stomach [49].

*E. tenuifolia* subsp. *sibthorpiana* (*E. sibthorpiana*), also known as tarhana herb or “Çörtük” in Turkish, is a popular aromatic plant in Turkey, the fresh or dried leaves of which are used as a flavoring agent in different foods such as meat and meatballs, pickles, and soups [67,75]. The leaves of the plant are used in pickles to prevent foaming and improve the shelf life of pickled products [76]. In some regions of Turkey, the young stems of *E. sibthorpiana* and *E. tournefortii* are eaten as a snack after pealing the outer part off the plant [77,78]. One of the main culinary uses of *E. sibthorpiana* is its use as a flavoring agent in the fermented cereal food tarhana, which is produced by mixing flour, yogurt, yeast, different vegetables (tomatoes, red peppers, etc.), and herbs (thyme, dill, tarhana herb, etc.), followed by fermentation for several days, drying and grinding, and then prepared as a soup [79,80,81]. Apart from flavoring, it has been found that the addition of appropriate ratios of *E. tenuifolia* subsp. *sibthorpiana* in tarhana could aid the fermentation process by preventing the decrease in yeast and lactic acid bacteria and improve the nutritional properties [67,82]. For medicinal purposes, the plant has been employed in the treatment of various conditions for its antispasmodic, digestive, galactagogue, and wound-healing properties [83,84,85]. Moreover, it is used orally in respiratory conditions like shortness of breath and externally for perspiration in case of the common cold [86,87]. Along with *E. tournefolii* (also known as “Dikenli Çörtük” in Turkish), it is used in the treatment of gastric ulcers in the form of 5% infusions [61,88].

While there is no record of traditional medical use of *E. spinosa*, the leaves and roots of the plant are recognized as edible, and the young shoots and leaves without thorns can be consumed pickled, in salads, or cooked [7]. The roots of *E. orientalis* are used as a flavoring agent and to impart tenderness to the traditional Turkish dessert “halva” (or “helva”) [65,78].

**Table 3:** Reported ethnomedicinal uses of *Echinophora* species.

Plant	Plant Part	Preparation	Uses	Ref.
*E. platyloba*	Aerial parts	Infusion, decoction	Kidney stones, cough, mouth ulcers (aphthae) (as a mouthwash)	[69]
		Decoction	Hepatoprotective	[70]
		Unspecified	Kidney pain	[74]
			Antifungal	[68]
			Anti-diarrheal	[71]
	Shoots	Infusion, decoction	Ileus, hemorrhoid, flatulence, joint pain	[72]
	Leaves	Extraction (unspecified procedure)	Common cold	[73]
*E. cinerea*	Aerial parts	Unspecified	Stimulant, stomach invigorator	[49]
*E. tenuifolia* subsp. *sibthorpiana*	Aerial parts	Infusion	Antispasmodic, digestive	[83]
		Decoction	Shortness of breath	[86]
		Unspecified	Hand, foot, and mouth wounds	[84]
		Infusion	Galactagogue	[85]
		Pounded	Externally for perspiration in case of colds	[87]
*E. tournefolii*	Flowering and leafy shoots	Infusion	Gastric ulcer	[88]

### 2.3. Pharmacological Activities of the Echinophora Genus

Extracts and EOs isolated from this genus exhibit a wide range of biological and pharmacological activities, among which antioxidant, antibiotic and antifungal, anti-inflammatory, anticancer, and insecticidal and larvicidal activities. An overview of the results from studies investigating these activities is presented in Table 4.

#### 2.3.1. Antibacterial Activity

The antibacterial activity of the aerial parts EO and different extracts (methanol, ethanol, and aqueous) from aerial parts and roots from *E. tenuifolia* subsp. *sibthorpiana* was investigated against eight pathogenic bacteria [6]. The EO demonstrated the highest antibacterial activity, specifically against *S. typhimurium* (MIC of 0.34 mg/mL, MBC of 1.35 mg/mL), *B. cereus* (MIC of 0.45 mg/mL, MBC of 1.35 mg/mL), and *P. aeruginosa* (MIC of 0.34 mg/mL, MBC of 2.70 mg/mL) [6]. In addition, strong antibacterial activity was observed with the ethanol root extract, which showed high activity against *B. cereus* (MIC of 0.45 mg/mL), as well as activity against *E. cloacae*, *E. coli*, *M. flavus*, *P. aeruginosa*, and *S. typhimurium* (with MIC of 1.5 mg/mL for all strains), while the aqueous extracts were the least active [6]. 

The EO isolated from *E. tenuifolia* subsp. *sibthorpiana* leaves was screened for antibacterial activity against thirteen bacterial strains, including Gram-positive (*S. aureus*, *S. epidermidis*, *S. warneri*, *S. hominis*, *B. cereus*, *E. faecalis*, *Streptococcus* spp.) and Gram-negative bacteria (*E. coli*, *S. flexneri*, *Enterobacter* spp., *Salmonella* spp., *Klebsiella* spp.) [39]. In general, the EO was more effective against the Gram-positive pathogens and demonstrated good antibacterial activity against *B. cereus* (MIC 62.5 μg/mL) and moderate activity against *S. epidermidis* and *S. aureus* (MIC 125 μg/mL for both strains), while the activity against the tested Gram-negative bacteria was weak [39]. Moreover, according to the composition of the studied EO, it could have a great potential for skin recovery effects [39]. One of the main compounds identified in the EO is α-phellandrene. This compound promotes the wound healing processes, accelerates the wound closure, and acts as an adhesive of primary intention [89]. α-Phellandrene contributes to collagen deposition as well [89]. The EO is rich in other compounds that are associated with wound-healing activity. These effects, in addition to the antibacterial activity, make the EO worth studying as a skin recovery therapy in surgical procedures or other interventions [89,90,91,92]. Currently there are no human studies in surgery for the wound healing activity of the EO isolated from *Echinophora* species.

Hashemi et al. investigated the antimicrobial activity of *E. platyloba* aerial parts EO and methanolic extract against food-borne pathogenic bacteria, including *S. aureus*, *L. monocytogenes*, *S. typhimurium*, and *E. coli* [57]. The results of the study revealed that while both the EO and the extract possessed antibacterial activity, the activity of the EO was higher than that of the methanolic extract [57]. The EO showed the highest activity against *L. monocytogenes* (MIC = MBC = 6250 ppm), as well as activity against *S. aureus* (MIC 12,500 ppm and MBC 25,000 ppm) and *E. coli* (MIC 50,000 ppm), whereas the extract was active only against *L. monocytogenes* and *S. aureus* (MIC 25,000 ppm for both strains). Neither the EO nor the extract showed activity against *S. typhimurium* [57].

Aqueous and ethanolic extracts from *E. platyloba* were screened for antibacterial activity against *A. faecalis*, *S. marcescens*, *P. rettgeri*, and *L. monocytogenes* [93]. Both extracts showed activity against the tested microorganisms [93]. The aqueous extract demonstrated the highest activity against *A. faecalis* (MIC = MBC = 31.25 mg/mL) and *L. monocytogenes* (MIC = 31.25 mg/mL and MBC = 62.5 mg/mL), while the ethanol extract was most effective against L. monocytogenes (MIC = MBC = 31.25 mg/mL) [93].

Ghasemi Pirbalouti and Gholipour reported the antibacterial activity of EO isolated from different populations of *E. cinerea* collected during different harvest times (early and full flowering stage) on five pathogenic microorganisms, including the Gram-positive bacteria *B. cereus* and *L. monocytogenes* and the Gram-negative bacteria *P. vulgaris* and *S. typhimurium* [49]. The EOs showed moderate-to-good antibacterial activity, especially against *B. cereus*, with the lowest MIC (32 μg/mL) for EO isolated from plants collected during the full flowering stage [49]. The same EO also showed highest activity against *L. monocytogenes* and *P. vulgaris* with MIC = 62 μg/mL [49].

The antibacterial activity of methanol extracts from different parts (leaves and ripe fruits) of *E. spinosa* against *B. subtilis*, *S. aureus*, *E. coli*, *P. mirabilis*, and *P. aeruginosa* was investigated by Ghadbane et al. [9]. The highest activity demonstrated the ripe fruit extract compared to leaves extract and gentamicin against *B. subtilis*, *E. coli*, and *P. mirabilis* with inhibition zones of 23.67 ± 1.53 mm, 30.50 ± 0.50 mm, and 20.50 ± 0.50 mm, respectively [9]. Neither the leaves nor ripe fruit methanol extracts showed activity against *S. aureus* and *P. aeruginosa* [9]. In addition, the EO isolated from ripe fruits of *E. spinosa* exhibited higher antibacterial activity compared to aerial parts EO in a study conducted by Fraternale et al. [8]. Both EOs inhibited the growth of potentially pathogenic Gram-positive gastrointestinal bacteria, including *C. difficile*, *C. perfringes*, *E. faecalis*, and *E. limosum*, with MIC values for the ripe fruit EO of 0.13% (*v*/*v*) [8]. Moreover, the MIC values were significantly higher for the valuable intestinal microflora tested, including two strains of *Bifidobacterium* and two strains of *Lactobacillus* (>4.00%) [8].

#### 2.3.2. Antifungal Activity

Aerial parts EO from *E. tenuifolia* subsp. *sibthorpiana* demonstrated strong antifungal properties against *A. versicolor*, *P. funiculosum*, *P. ochrochloron* with MIC values of 0.17 mg/mL, and *A. fumigatus*, *A. ochraceus*, *T. viride* with MIC values of 0.34 mg/mL, while its activity against *C. albicans* was lower (MIC 2.70 mg/mL) [6]. The antifungal activities of the root and aerial parts ethanol, methanol, and aqueous extracts of the same plant were significantly lower than that of the EO, with aqueous extracts exhibiting the lowest antifungal activity [6]. Methanol extract from the ripe fruits of *E spinosa* demonstrated better activity than leaf extract against *C. albicans* with an inhibition zone of 19.67 ± 1.53 mm [9]. 

Out of all *Echinophora* species, the most promising antifungal activity has been reported for *E. platyloba*, which has been an object of numerous in vitro studies demonstrating its antifungal potential, linking it to its ethnomedicinal use [66,94].

The antifungal activity of ethanolic extract of *E. platyloba* was investigated against the dermatophytes *T. schenlaini*, *T. verucosum*, *T. rubrum*, *M. gypsum*, *T. violaseum*, *T. mentagrophytes*, *M. canis* and *E. flucosum* and the extract exhibited the highest activity against *T. schenlaini* and *T. verucosum* at concentrations as low as 35 mg/mL [66,94]. Consequently, the activity of the extract was screened against *C. albicans*. The extract showed significant anti-*Candida* activity at different concentrations, the lowest concentration inducing inhibition of the growth being 2 mg/mL [95].

Ethanol and ethyl acetate extracts from *E. platyloba* leaves showed good antifungal activity against nine clinically isolated strains of *C. albicans*, with the lowest observed MIC (12.5 mg/mL) for the ethyl acetate extract [96]. Moreover, extract from *E. platyloba* not only inhibited the growth of clinical strains of *C. albicans* resistant to fluconazole, but it also reduced the expression of the CDR1 and CDR2 genes, which play an important role in the development of azole drug resistance [97].

In a study investigating the synergistic anti-*Candida* activity of ethanolic extract of *E. platyloba* and azole antimycotics, it was found that the *E. platyloba* extract exhibited synergism with fluconazole and itraconazole and antagonism with clotrimazole and miconazole against clinical strains of *C. albicans* [98]. The activity of the combination of fluconazole with *E. platyloba* extract was assessed in a randomized double-blind clinical trial, which included sixty women with recurrent candidal vaginitis [99]. The participants were randomized into two groups, one of which was treated with fluconazole alone and the other treated with fluconazole and cream containing *E. platyloba* extract. The group receiving fluconazole and *E. platyloba* cream showed a statistically significant decrease in positive culture results after fourteen days of treatment and a decrease in the frequency of recurrence of the vaginitis [99].

In addition to fluconazole and itraconazole, the extract showed synergism with amphotericin B [94]. The combination of 5% ethanolic extract with amphotericin B showed an increase in the zone of inhibition by 22% compared to amphotericin B alone (22 mm for the combination compared to 18 mm for amphotericin B alone) and a decrease in the MIC by 50% (1 mg/mL for the combination compared to 2 mg/mL for amphotericin B alone) [94].

#### 2.3.3. Anti-Parasitic Activity

Ngahang Kamte et al. examined the activity of the EOs isolated from nine Apiaceae species, including *E. spinosa* root and aerial parts EOs, and their major compounds against the protozoan *Trypanosoma brucei*, the causative agent of African sleeping sickness [33]. The *E. spinosa* root EO demonstrated the highest trypanocidal activity among the tested EOs (EC_50_ value of 2.7 ± 0.6 μg/mL), while the highest activity out of all tested samples was observed with terpinolene (EC_50_ value of 0.035 ± 0.005 μg/mL), which was found to be one of the major constituents of the EO [33].

#### 2.3.4. Insecticidal and Larvicidal Activity

**Figure 3:**
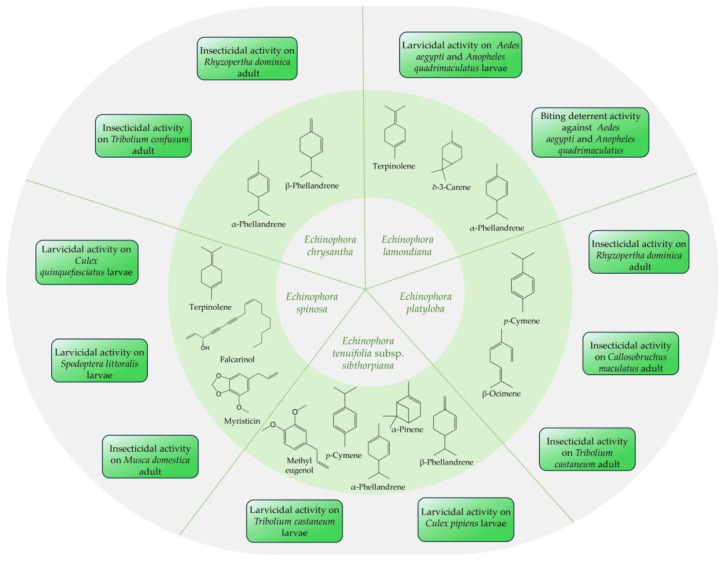
Insecticidal, larvicidal, and biting deterrent activities of *Echinophora* EOs.

The EOs isolated from *Echinophora* species demonstrated promising insecticidal and larvicidal properties underlying their potential use as biopesticides and insect repellents (Figure 3) [7,45,59,62,65].

In a study conducted by Papanikolaou et al., the biopesticide potential of microemulsion formulations of six EOs, including *E. tenuifolia* subsp. *sibthorpiana* EO, was assessed against two beetle species (*Trogoderma granarium* and *Tribolium castaneum*) in different development stages (larvae and adult) [45]. The microemulsion of the *E. tenuifolia* subsp. *sibthorpiana* EO showed significant activity against *T. castaneum* larvae with a mean mortality rate of 90.0% fourteen days post-exposure, while its activity against *T. castaneum* adults and *T. granarium* was lower [45]. Additionally, in a study by Evergetis et al., *E. tenuifolia* subsp. *sibthorpiana* EO demonstrated good activity against larvae of the *Culex pipiens* mosquitoes with LC_50_ values of 59.46 mg/L [46].

The insecticidal and larvicidal activity of *E. spinosa* EO was investigated in two studies by Pavela et al. [7,34]. In the first study, the larvicidal activity of the EOs isolated from aerial parts and roots of *E. spinosa* was assessed against the larvae of the medically important vector *Cx. quinquefasciatus*. The root EO demonstrated high efficacy against the *Cx. quinquefasciatus* larvae with LC_50_ values of 18.9 μL/L, which was significantly higher than that of the aerial parts EO (LC_50_ of 40.5 μL/L) [34]. These results were confirmed in a second study in which the root EO showed higher activity than the leaves and stems EO against *Cx. quinquefasciatus* larvae (LC_50_ of 15.7 μL/L for the root EO compared to LC_50_ of 41.3 μL/L for the aerial parts EO) [7]. Moreover, the root EO demonstrated relevant insecticidal and larvicidal activity against *Musca domestica* adults (LD_50_ of 38.3 μg/adult) and *Spodoptera littoralis* larvae (LD_50_ of 55.6 μg/larvae) [7].

Comparison between the larvicidal and biting deterrent activities of EOs isolated from different parts (stems, leaves, and flowers) of *E. lamondiana* and their major constituents (δ-3-carene, α-phellandrene, and terpinolene) was conducted by Ali et al. [65]. The leaf EO was significantly more toxic than the stem and flower EOs and exhibited the highest larvicidal activity against *An. quadrimaculatus* larvae with LC_50_ of 26.5 ppm, whereas its activity against *Ae. aegypti* was lower (LC_50_ of 138.3 ppm) [65]. In addition, the flower and leaf EOs showed biting deterrent activity against *Ae. aegypti* and *An. quadrimaculatus* at 10 mg/cm^2^, similar to the insect repellent N,N-diethyl-meta-toluamide (DEET) at 25 nmol/cm^2^ [65].

The EO obtained from aerial parts of *E. chrysantha* showed dose- and time-dependent contact insecticidal activity on *Rhyzopertha dominica* and *Tribolium confusum* [62]. The highest activity was observed against *T. confusum*, with a mortality rate of 37.9% after 48 h of exposure, which was higher than that of *R. dominica* (25.8%) [62]. 

The fumigant and contact toxicity of EO from the aerial parts of *E. platyloba* on three beetle species (*T. castaneum*, *R. dominica*, and *Callosobruchus maculatus*) was demonstrated in a study by Sharifian and Darvishzadeh [59]. The EO showed significant dose- and time-dependent fumigant toxicity against *R. dominica* and *C. maculatus* with LC_50_ values of 5.66 and 3.835 μL/250 mL air after 24 h, respectively, while the highest contact insecticidal activity was observed with *R. dominica* with mean LC_50_ values of 9.712 μL/39 cm^2^ [59]. In both cases, the most resistant species was *T. castaneum* [59]. Additionally, the aerial parts EO of *E. platyloba* showed very low toxicity on *Encarsia formosa*, a parasitic wasp used in the biocontrol of the pest *Trialeurodes vaporariorum* (greenhouse whitefly) [100].

#### 2.3.5. Anticancer Activity

Marrelli et al. examined the antiproliferative activity of methanol extract from *E. tenuifolia* branches and its hexane, ethyl acetate, and dichloromethane fractions on three human cancer cell lines, namely, colorectal adenocarcinoma (LoVo), melanoma (C32) and breast cancer (SKBr3) [11]. All the tested samples showed significant inhibition on the cell proliferation with a percentage range between 55.6 ± 0.7 and 97.8 ± 0.9%, the highest percentages being for dichloromethane fraction on melanoma cancer cells (97.8 ± 0.9% with IC_50_ values of 22.8 ± 0.8 μg/mL) and colorectal adenocarcinoma cells (83.5 ± 3.8% with IC_50_ values of 53.0 ± 2.1 μg/mL) [11].

Methanolic extracts of *E. platyloba* showed promising anticancer activities on various cancer cell lines, including prostate adenocarcinoma, fibrosarcoma, breast cancer, and leukemia cell lines [12,101,102,103]. The methanolic extract from aerial parts of *E. platyloba* decreased cell viability and induced apoptotic cell death in malignant prostate adenocarcinoma (PC 3) cell line with IC_50_ values of 236.136 ± 12.4, 143.400 ± 7.2, and 69.383 ± 1.29 μg/mL after 24, 36, and 48 h, respectively, while it demonstrated no significant activity on non-malignant Human Umbilical Vein Endothelial Cells HUVEC cell line [12]. Moreover, apoptotic cell death and suppression of cell proliferation were induced by *E. platyloba* extract in mouse fibrosarcoma cell line (WEHI-164) [101]. The effects of *E. platyloba* methanolic extract on a human breast cancer cell line (MDA-MB-231) were investigated by Birjandian et al. [102]. The extract decreased the viability and induced apoptotic cell death on MDA-MB-231 cells, with the highest activity being observed in a concentration of 534.6 ± 7.2 μg/mL after 24 h [102]. The methanol leaf extract of *E. platyloba* revealed an antimutagenic effect and significantly inhibited the proliferation of Acute Promyelocytic Leukemia cell line (NB4) with the highest activity of 87.35% at a concentration of 500 μg/mL after 24 h [103].

Akşit et al. screened the antiproliferative activity of *E. chrysantha* ethanol extract on various malignant cell lines, including brain cancer, gynecological cancer, and colon cancer cell lines [24]. The extract demonstrated the highest activity against HT-29 colon cancer cells with IC_50_ of 4.07 ± 0.2 μg/mL and HeLa gynecological cancer cells with IC_50_ of 1.41 ± 0.1 μg/mL, while the observed cytotoxicity against normal cell lines (lung, retinal, and skin) was low [24].

In a study by Amirghofran et al., the activity of methanol extract from the aerial parts of *E. cinerea* was screened against four cancer cell lines, including lung carcinoma (A549) cell line, bladder carcinoma (Fen) cell line, myelogenous leukemia (K562) cell line, and T cell leukemia (Jurkat) cell line [104]. The extract exhibited strong antiproliferative effects on the Jurkat T cell leukemia cell line (IC_50_ of 6.9 μg/mL), as well as significant antiproliferative effects on the K562 myelogenous leukemia cell line [104].

**Figure 4:**
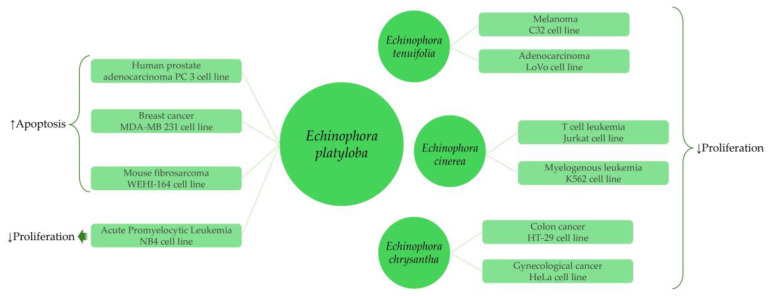
Anticancer effects of *Echinophora* extracts on different cancer cell lines. Downward arrows indicate inhibition, while upward arrows indicate stimulation.

The anticancer effects of *Echinophora* species on different cancer cell lines are summarized in Figure 4.

#### 2.3.6. Anti-Inflammatory Activity

The in vitro anti-inflammatory activity of *E. tenuifolia* inflorescence methanol extract was demonstrated in a study by Marrelli et al. [19]. The n-hexane and dichloromethane fractions of the extract exhibited significant anti-inflammatory activity by inhibiting the lipopolysaccharide-induced production of NO in macrophages (RAW 264.7 cell line) with IC_50_ values of 17.04 ± 1.37 μg/mL and 39.97 ± 3.16 μg/mL, respectively, which were lower than that of the positive control indomethacin (58.00 ± 0.9 μg/mL) [19].

Chianese et al. reported the modulation of six transient receptor potential (TRP) proteins by the polyacetylenes echinophorin A, echinophorin B, and echinophorin D isolated from *E. platyloba* extract. The three polyacetylenes exhibited anti-inflammatory activity by selective action toward the TRP ankyrin 1 (TRPA1) cation channel, an ion channel that plays a role in the mediation of neuropathic and inflammatory pain [2].

#### 2.3.7. Analgesic Effects

The analgesic effects of *E. platyloba* leaf methanolic extract were investigated in vivo on male Wistar rats using three different tests (tail flick, rating, and formalin tests) in a study by Asgari Nematian and Mohammadi [105]. In both acute and chronic phases, the extract produced significant analgesic activity and reduced the pain peripherally and centrally, the highest activity being observed with the highest tested dose of 300 mg/kg [105]. Furthermore, low doses of the ethanolic extract from the aerial parts of *E. platyloba* induced rewarding effects in vivo in female albino mice, most likely due to effects on the opioid receptors, and inhibited the rewarding effects of morphine [106].

In a single-blind clinical study involving sixty women with dysmenorrhea, *E. platyloba* extract showed considerably higher analgesic activity compared to placebo [107,108]. 

#### 2.3.8. Hormonal Effects

The effectiveness in the treatment of moderate-to-severe premenstrual syndrome (PMS) with *E. platyloba* extract was assessed by two clinical studies [15,109]. The first study, a single-blind randomized clinical trial, included ninety women with moderate-to-severe PMS, who were randomized into three groups: a group receiving *E. platyloba* extract, a group receiving fennel extract, and a placebo group. A reduction in the intensity of the PMS symptoms was observed in all three groups, yet the extracts showed a more significant reduction in the severity of the PMS [15]. In a second single-blind randomized clinical trial, sixty women with moderate-to-severe PMS were randomized into two groups: a group receiving *E. platyloba* extract and a placebo group. There was a significant reduction in the PMS symptoms in the *E. platyloba* group after the treatment, especially in the anxiety and depression symptoms [109]. 

In an in vivo study on male Wistar rats, the effects of *E. platyloba* aerial parts alcoholic extract on the levels of prolactin and on the activity of the pituitary–gonadal axis were described [110]. In the animals treated with the *E. platyloba* extract, a significant increase in the levels of testosterone and prolactin levels was observed, in addition to a significant decrease in cholesterol levels [110]. 

#### 2.3.9. Organoprotection and Cytoprotection

Heidarian et al. investigated the hepatoprotective effect of *E. platyloba* leaf ethanol-water extract in an in vivo acetaminophen-induced hepatotoxicity model in rats [111]. The *E. platyloba* extract showed excellent hepatoprotective effects characterized by a reduction in the serum levels of hepatic enzymes like the transaminase enzymes aspartate transaminase (AST) and alanine transaminase (ALT), induction of the liver catalase (CAT) activity and antioxidant capacity of the liver and histopathological improvements [111]. These results support the traditional medicinal use of *E. platyloba* as a hepatoprotective agent [70].

Ethanolic extract from leaves of *E. cinerea* demonstrated promising cardioprotective effects [14]. The effect of the extract on cardiac function was assessed in vivo in rats with aluminum phosphide-induced poisoning. The *E. cinerea* extract improved the hemodynamic and the electrocardiogram parameters caused by the poisoning, including an increase in the arterial systolic blood pressure, prevention of the decline in heart rate, and increase in the corrected QT interval [14]. 

In a study by Shokoohinia et al., two compounds isolated from acetone extract of *E. cinerea*, namely quercetin-3-*O*-b-D-glucopyranoside and osthol, showed in vitro protective effects against cisplatin-induced cytotoxicity in rat pheochromocytoma-derived PC12 cell line, often used as a model for dopaminergic neurons [20]. The two molecules exerted their effect by inhibiting apoptosis. The results from the study demonstrate the potential use of *E. cinerea* in the prevention of cisplatin-induced neurotoxicity [20]. In addition, quercetin-3-*O*-b-D-glucopyranoside suppressed the generation of reactive oxygen species and exhibited cytoprotective effects on the PC12 cell line in H_2_O_2_-induced cytotoxicity [21].

**Table 4:** Biological and pharmacological activities of the *Echinophora* genus.

Plant	Plant Part	Extract/Fraction	Activity	Effect	Ref.
*E. tenuifolia*	Inflorescences	Methanol/Ethyl acetate fraction	Antioxidant	Showed high antioxidant activity in DPPH and β-carotene bleaching assays (IC50 40.39 ± 0.41 μg/mL and 46.66 ± 0.82 μg/mL after 60 min, respectively)	[19]
		Methanol/n-Hexane fraction	Anti-inflammatory	Exhibited NO inhibitory effect (IC_50_ 17.04 ± 1.37 μg/mL) and induced low cytotoxicity (IC_50_ 94.45 ± 3.24 μg/mL)	
		Methanol/Dichloromethane fraction		Exhibited NO inhibitory effect (IC_50_ of 39.97 ± 3.16 μg/mL and showed no cytotoxicity (IC_50_ values >200 μg/mL)	
	Branches	Methanol/Dichloromethane fraction	Antiproliferative	Showed high antiproliferative activity against melanoma cancer cell line C32 (IC_50_ 22.8 ± 0.8 μg/mL) and LoVo adenocarcinoma cell line (IC_50_ 53.0 ± 2.1 μg/mL)	[11]
*E. tenuifolia* subsp. *sibthorpiana*	Leaves	Essential oil	Antibacterial	Showed good antibacterial activity against *Bacillus cereus* (MIC 62.5 μg/mL) and moderate activity against *Staphylococcus epidermidis* and *Staphylococcus aureus* (MIC 125 μg/mL for both strains)	[39]
	Aerial parts		Insecticidal and larvicidal	Microemulsion of the EO showed a 90.0% mean mortality rate against *Tribolium castaneum* larvae	[45]
				Showed high activity against *Culex pipiens* larvae with LC_50_ values of 59.46 mg/L	[46]
			Antifungal	Showed a potent antifungal activity on *Rhizoctonia solani* (mean growth inhibition percentage of over 80%)	[44]
				Showed a potent antifungal activity against *Aspergillus ochraceus* and *Penicillium ochrochloron* (MIC 0.17 mg/mL and MFC 0.34 mg/mL for both strains)	[6]
			Antibacterial	Showed a potent antibacterial activity (MIC in the range of 0.67 to 2.70 mg/mL); highest activity against *Bacillus cereus* and *Salmonella typhimurium* (MIC 0.67 mg/mL and MBC 1.35 mg/mL for both strains)	
		Ethanol	Antifungal	Showed an antifungal activity against *Aspergillus versicolor* (MIC 1.25 mg/mL and MFC 2.50 mg/mL)	
	Roots		Antibacterial	Showed a potent antibacterial activity against *Bacillus cereus* (MIC 0.45 mg/mL and MBC 0.75 mg/mL)	
*E. platyloba*	Stems	Water	Antibacterial	Exhibited highest activity against *Alcaligenes faecalis* (MIC = MBC = 31.25 mg/mL)	[93]
		Ethanol		Exhibited highest activity against *Listeria monocytogenes* (MIC = MBC = 31.25 mg/mL)	
	Aerial parts		Antifungal	Revealed an antifungal activity on standard strain (MIC 32 mg/mL and MFC 64 mg/mL) and clinical isolates (MIC 64 mg/mL and MFC 128 mg/mL) of *Candida albicans*	[97]
				Revealed an antifungal activity against clinical isolates of *C. albicans* (MIC 3.1–6.25 mg/mL and MFC 6.2–12.5 mg/mL) and synergistic antifungal activity with fluconazole and itraconazole	[98]
		Unspecified	Antibacterial	Exhibited activity against *Salmonella enteritidis* (MIC 50 mg/mL, MBC 150 mg/mL, and zone inhibition of 26.11 ± 1.16 mm)	[16]
		Essential oil		Exhibited activity against *Listeria monocytogenes*, *Staphylococcus aureus*, and *Escherichia coli* (MIC 6250, 12,500, and 50,000 ppm, respectively)	[57]
		Methanol		Exhibited activity against *Listeria monocytogenes* and *Staphylococcus aureus* (MIC 25,000 ppm for both pathogens)	
			Anticancer	Showed a decrease in cell viability in the human prostate adenocarcinoma PC 3 cell line (IC_50_ 236.136 ± 12.4, 143.400 ± 7.2, and 69.383 ± 1.29 μg/mL after 24, 36, and 48 h, respectively)	[12]
				Showed inhibition of the proliferation of mouse fibrosarcoma WEHI-164 cell line (IC_50_ 196.673 ± 12.4 μg/mL)	[101]
	Leaves			Possessed activity against breast cancer MDA-MB-231 cell line (IC_50_ 534.6 ± 7.2 μg/mL during 24 h)	[102]
				Showed significant inhibition of the proliferation of Acute Promyelocytic Leukemia NB4 cell line (highest activity of 87.35% after 24 h at a concentration of 500 μg/mL)	[103]
			Analgesic	Exhibited significant analgesic activity in vivo via tail flick, rating, and formalin tests on male Wistar rats with a dose of 300 mg/kg	[105]
	Aerial parts	Ethanol:Water 80:20	Hepatoprotective	Demonstrated hepatoprotective effect in acetaminophen-induced hepatotoxicity in rats treated with 200, 500, and 1000 mg/kg of the extract	[111]
*E. cinerea*	Leaves	Ethanol	Cardioprotective	Showed improvement in bradycardia, hypotension, and cardiac conduction in aluminum phosphide-induced cardiotoxicity in rats at a dose of 200 mg/kg	[14]
	Aerial parts	Methanol	Anticancer	Exhibited strong antiproliferative effect on T cell leukemia Jurkat cell line (IC_50_ 6.9 μg/mL) and cytotoxic effects on myelogenous leukemia K562 cell line	[104]
			Immunomodulatory	Exhibited significant reduction in the proliferation of lymphocytes in concentrations from 10 to 200 μg/mL	[112]
		Essential oils	Antibacterial	Demonstrated moderate-to-good antibacterial activity against *B. cereus* (MIC 32 μg/mL and MBC 125 μg/mL) and *L. monocytogenes* (MIC 62 μg/mL and MBC 125 μg/mL)	[49]
*E. spinosa*	Leaves	Methanol	Antimicrobial	Exhibited highest activity against *Bacillus subtilis* and *E. coli* (inhibition zones of 13.67 ± 1.5 mm and 13.50 ± 0.87 mm, respectively)	[9]
	Fruits			Exhibited higher activity compared to leaves extract against *Bacillus subtilis*, *E. coli*, and *C. albicans* (inhibition zones of 23.67 ± 1.5 mm, 30.50 ± 0.50 mm, and 19.67 ± 1.53 mm, respectively)	
		Essential oil		Demonstrated selective antibacterial activity against potentially pathogenic intestinal bacteria *Clostridium difficile*, *C. perfringes*, *Enterococcus faecalis*, and *Eubacterium limosum* (MIC = 0.13% (*v*/*v*)) compared to valuable microflora like *Bifidobacterium* and *Lactobacillus* (MIC > 4.00%)	[8]
			Cytotoxic	Showed significant cytotoxic effect on human promonocytoid U937 cell culture (IC_50_ 14.5 ± 0.85 μg/mL)	[113]
	Roots		Aantitrypanosomal	Exhibited good inhibitory effects on the protozoan *Trypanosoma brucei* (EC_50_ 2.7 ± 0.6 μg/mL), highest among the tested EO	[33]
			Insecticidal and larvicidal	Demonstrated significant toxic effects on *Culex quinquefasciatus* larvae (LC_50_ 15.7 mg/L) in addition to toxic effect against *Musca domestica* (adult) and *Spodoptera littoralis* (larvae) (LD_50_ 38.3 μg/adult and 55.6 μg/larva, respectively)	[7]
				Demonstrated significant toxic effects on *C. quinquefasciatus* larvae with (LC_50_ 18.9 μL/L)	[34]
*E. lamondiana*	Flowers	Essential oil	Biting deterrent	Produced biting deterrent activity against *Aedes aegypti* (L.) and *Anopheles quadrimaculatus* Say. at 10 μg/cm^2^, statistically similar to DEET at 25 nmol/cm^2^	[65]
	Leaves				
			Larvicidal	Showed larvicidal activity against *Ae. aegypti* and *An. quadrimaculatus* (LC_50_ 138.3 and 26.2 ppm, respectively)	
*E. chrysantha*	Aerial parts	Ethanol	Antiproliferative	Exhibited strong antiproliferative activity against HT-29 colon cancer cell lines (IC_50_ 4.07 ± 0.2 μg/mL) and HeLa gynecological cancer cell lines (IC_50_ 1.41 ± 0.1 μg/mL) with low cytotoxicity toward normal cell lines (IC_50_ ranging from 91.24 ± 4.0 to 118.03 ± 3.1 μg/mL)	[24]
			Antioxidant	Exhibited strong antioxidant activity in DPPH assay (IC_50_ 11.52 ± 1.83 μg/mL) and ferric reducing power assay (63.58 ± 4.05 µg TE/g)	
		Essential oil	Antibacterial	Showed activity against *L. monocytogenes*, *S. aureus*, and *Enterococcus faecium* (MIC 31.25, 31.25, and 62.5 μg/mL, respectively)	[62]
			Antifungal	Produced moderate inhibition of the mycelium growth of *Phytophthora infestans*, *Verticillium dahlia*, and *Fusarium oxysporum* f. sp. *lycopersici* at 8 μL/Petri	
			Insecticidal	Demonstrated moderate activity against two insects, *Rhyzopertha dominica* and *Tribolium confusum*, with mortality rates of 25.8 ± 0.6% and 37.9 ± 1.2% at a concentration of 5% (*v*/*v*)	

## 3. Materials and Methods

The search strategy was to screen for studies regarding the occurrence, isolation, and identification of phytochemical bioactive compounds in *Echinophora* species and their ethnomedicinal use and pharmacological activities. The studies were obtained from scientific electronic databases, including PubMed, Google Scholar, Scopus, Springer Link, and ScienceDirect. The keywords included in the search were: “bioactive compounds”, “*Apiaceae*”, “*Echinophora*”, “*Echinophora* extract”, “*Echinophora* essential oil”, “*Echinophora platyloba*”, “*Echinophora cinerea*”, “*Echinophora chrysantha*”, “*Echinophora spinosa*”, “*Echinophora tenuifolia*”, “*Echinophora lamondiana*”, “*Echinophora tournefortii*”, “*Echinophora tenuifolia* subsp. *sibthorpiana*”, “ethnomedicinal use”, “animal studies”, “cell culture studies”. The keywords were used separately or in combination, depending on the database. Articles not in English and not relevant to this review were excluded. A total of 113 articles were included in the review, 57 of which concerned the pharmacological and biological activities of the genus.

## 4. Conclusions

The species within the genus *Echinophora* are valued for their edible and medicinal properties, often used as flavoring agents, preservatives, and in the treatment of various health conditions. Although the genus is associated with various applications and safety, the scientific studies on *Echinophora* species remain limited. Plants belonging to this genus are rich in phenolic compounds, including flavonoids, phenolic acid, and coumarins, as well as volatile and non-volatile terpenes and polyacetylenes. The extracts and the EOs derived from *Echinophora* species are associated with significant therapeutic potential and have demonstrated a wide range of biological activities, including anticancer, antibacterial, antifungal, anti-inflammatory, hepatoprotective, and insecticidal. Among the *Echinophora* species, *E. platyloba* demonstrated the greatest antifungal activity. Studies about *E. platyloba* also reported excellent hepatoprotective, analgesic, and anticancer activities. Most of the studies related to the genus *Echinophora* were performed in preclinical settings (in vitro and in vivo), apart from four clinical studies with humans.

However, there is limited information on the secondary metabolites produced by these species and their pharmacological effects. Further research is required on the phytochemistry and pharmacology of the extracts and isolated chemical compounds from these plants. Metabolomic studies could be useful for gaining new insights into the phytochemistry, while more comprehensive in vitro and in vivo studies, as well as molecular docking studies, could be beneficial for the discovery of new potential pharmacologically active substances from the species within this botanical genus.

## Figures and Tables

**Table 1:** Compounds identified in extracts of *Echinophora* spesies.

Plant	Country of Origin	Main Phytochemicals	Refs.
*E. tenuifolia* (branches)	Italy	*o*-cymene, cumic alcohol, linoleic acid, myristic acid, 10,13-octadecadienoic acid, stigmasterol, carvacrol, rutin	[11]
*E. tenuifolia* (inflorescences)		myristic acid, palmitic acid, α-phellandrene, *o*-cymene, dihydroactinidiolide, neophytadiene, phytol, β-amyrin, carvacrol, ferulic acid, rutin, quercitrin	[19]
*E. cinerea* (aerial parts)	Iran	echinophorin A, echinophorin B, echinophorin C, verbenone-5-*O*-β-D-glycopyranoside, osthol	[17]
		quercetrin-3-*O*-β-D-glucopyranoside, osthol, verbenone-5-*O*-β-D-glycopyranoside, isoimperatorin, kaempferol-3-*O*-β-D-glucopyranoside, echinophorin B	[20,21]
*E. platyloba* (aerial parts)		echinophorin A, echinophorin B, echinophorin D, coriolic acid	[2]
		stigmasterol, sitosterol, stigmasterol-β-D-glycoside	[22]
		quercetin	[23]
*E. chrysantha* (aerial parts)	Turkey	flavonoids (hesperidin, luteolin-7-*O*-glucoside, quercetin-3- *O*-glucuronide, quercetin-3-*O*-glucoside, quercetin-3-*O*-rhamnoside, kaempferol-3-*O*-glucoside, kaempferol-3-*O*-rutinoside, rutin, quercetin, luteolin, naringenin, apigenin, and chrysin); organic acids (salicylic acid, quinic acid, fumaric acid, aconitic acid, *o*-coumaric acid, protocatechuic acid, chlorogenic acid, caffeic acid, syringic acid, *p*-coumaric acid, and sinapic acid)	[24]
*E. tournefortii*		2,5-dihydroxybenzoic, rutin, caffeic acid	[25]

## References

[1] Hosseini Z., Lorigooini Z., Rafieian-Kopaei M., Shirmardi H.A., Solati K. (2017). A Review of Botany and Pharmacological Effect and Chemical Composition of *Echinophora* Species Growing in Iran. Pharmacogn. Res..

[2] Chianese G., Sirignano C., Shokoohinia Y., Mohammadi Z., Bazvandi L., Jafari F., Jalilian F., Schiano Moriello A., De Petrocellis L., Taglialatela-Scafati O. (2018). TRPA1 Modulating C14 Polyacetylenes from the Iranian Endemic Plant *Echinophora platyloba*. Molecules.

[3] Genc I., Ecevit Genç G. (2014). The Synopsis of the *Genus echinophora* L. (Apiaceae) in Turkey. J. Fac. Pharm. Istanb. Univ..

[4] Christina G., Koutsaviti A., Bazos I., Tzakou O. (2010). Chemical Composition of *Echinophora tenuifolia* subsp. sibthorpiana Essential Oil from Greece. Rec. Nat. Prod..

[5] Ivanova S., Dyankov S., Karcheva-Bahchevanska D., Todorova V., Georgieva Y., Benbassat N., Ivanov K. (2023). *Echinophora tenuifolia* subsp. sibthorpiana—Study of the Histochemical Localization of Essential Oil. Molecules.

[6] Mileski K., Dzamic A., Ciric A., Grujić S., Ristić M., Matevski V., Marin P. (2014). Radical Scavenging and Antimicrobial Activity of Essential Oil and Extracts of *Echinophora sibthorpiana* Guss. from Macedonia. Arch. Biol. Sci..

[7] Pavela R., Maggi F., Cianfaglione K., Canale A., Benelli G. (2020). Promising Insecticidal Efficacy of the Essential Oils from the Halophyte *Echinophora spinosa* (Apiaceae) Growing in Corsica Island, France. Environ. Sci. Pollut. Res..

[8] Fraternale D., Genovese S., Ricci D. (2013). Essential Oil Composition and Antimicrobial Activity of Aerial Parts and Ripe Fruits of *Echinophora spinosa* (Apiaceae) from Italy. Nat. Prod. Commun..

[9] Ghadbane M. (2020). Antimicrobial and Antioxidant Activity of Methanol Extract of *Echinophora spinosa* L. from Jijel, Algeria. Alger. J. Biosci..

[10] El Mokni R. (2020). *Echinophora spinosa* L. (Apiaceae), a New Species in the Flora of Tunisia and Second Report from North Africa. Hacquetia.

[11] Marrelli M., Pisani F., Amodeo V., Duez P., Conforti F. (2020). *Echinophora tenuifolia* L. Branches Phytochemical Profile and Antiproliferative Activity on Human Cancer Cell Lines. Nat. Prod. Res..

[12] Shahneh F.Z., Baradaran B., Majidi J., Babaloo Z. (2014). *Echinophora platyloba* DC (Apiaceae) Crude Extract Induces Apoptosis in Human Prostate Adenocarcinoma Cells (PC 3). Biomed. J..

[13] Kalantari M., Entezari M., Movafagh A., Hushmandi K., Dehghani H. (2021). Apoptotic Genes of Bax, Bad, Bcl2, and P53 in A549 Lung Cancer Cells Comparison of the Effect of *Echinophora platyloba* DC. Extract and *Cordia myxa* L. Extract on the Expression of Apoptotic Genes of Bax, Bad, Bcl2, and P53 in A549 Lung Cancer Cells. Gulf J. Oncol..

[14] Haydari S., Nazari A., Moghimian M., Sedighi M., Ghaderpour S. (2020). Cardioprotective Activity of Ethanolic Extract of *Echinophora cinerea* against Aluminum Phosphide Poisoning in Rats. J. Food Biochem..

[15] Delaram M., Kheiri S., Hodjati M.R. (2011). Comparing the Effects of Echinophora-Platyloba, Fennel and Placebo on Pre-Menstrual Syndrome. J. Reprod. Infertil..

[16] Ranjbar R., Babaie S. (2016). Evaluation the Antibacterial Effects of *Echinophora platyloba* Extracts against Some Salmonella Species. Electron. Physician.

[17] Jelodarian Z., Shokoohinia Y., Rashidi M., Ghiasvand N., Hosseinzadeh L., Iranshahi M. (2017). New Polyacetylenes from *Echinophora cinerea* (Boiss.) Hedge et Lamond. Nat. Prod. Res..

[18] Sousa R.M.O.F., Cunha A.C., Fernandes-Ferreira M. (2021). The Potential of Apiaceae Species as Sources of Singular Phytochemicals and Plant-Based Pesticides. Phytochemistry.

[19] Marrelli M., Statti G.A., Menichini F., Conforti F. (2017). *Echinophora tenuifolia* L. Inflorescences: Phytochemistry and In Vitro Antioxidant and Anti-Inflammatory Properties in LPS-Stimulated RAW 264.7 Macrophages. Plant Biosyst.-Int. J. Deal. All Asp. Plant Biol..

[20] Shokoohinia Y., Khajouei S., Ahmadi F., Ghiasvand N., Hosseinzadeh L. (2017). Protective Effect of Bioactive Compounds from *Echinophora cinerea* against Cisplatin-Induced Oxidative Stress and Apoptosis in the PC12 Cell Line. Iran. J. Basic. Med. Sci..

[21] Shokoohinia Y., Rashidi M., Hosseinzadeh L., Jelodarian Z. (2015). Quercetin-3-*O*-*β*-d-Glucopyranoside, a Dietary Flavonoid, Protects PC12 Cells from H_2_O_2_-Induced Cytotoxicity through Inhibition of Reactive Oxygen Species. Food Chem..

[22] Valizadeh H., Mahmoodi F., Alizadeh Z., Bahadori M.B. (2014). Isolation and Structure Elucidation of Secondary Metabolites from *Echinophora platyloba* DC from Iran. J. Med. Plants.

[23] Hadjmohammadi M., Karimiyan H., Sharifi V. (2013). Hollow Fibre-Based Liquid Phase Microextraction Combined with High-Performance Liquid Chromatography for the Analysis of Flavonoids in *Echinophora platyloba* DC. and Mentha Piperita. Food Chem..

[24] Aksit Z., Aksit H., Şimşek S. (2022). LC-MS/MS Profiling Phytochemical Content of *Echinophora chrysantha* (Apiaceae) Andantiproliferative, Antioxidant Activity. Hosp. Pharmacol. Int. Multidiscip. J..

[25] Kaska A. (2021). The screening of *Echinophora tournefortii* Jaup & Spach for toxicity, antioxidant properties, phenolic profile and mineral elements. Fresenius Environ. Bull..

[26] Berga M., Logviss K., Lauberte L., Paulausks A., Mohylyuk V. (2023). Flavonoids in the Spotlight: Bridging the Gap between Physicochemical Properties and Formulation Strategies. Pharmaceuticals.

[27] Christensen L.P., Brandt K. (2006). Bioactive Polyacetylenes in Food Plants of the Apiaceae Family: Occurrence, Bioactivity and Analysis. J. Pharm. Biomed. Anal..

[28] Jiang Y.-P., Liu Y.-F., Guo Q.-L., Jiang Z.-B., Xu C.-B., Zhu C.-G., Yang Y.-C., Lin S., Shi J.-G. (2015). C14-Polyacetylene Glucosides from Codonopsis Pilosula. J. Asian Nat. Prod. Res..

[29] Zhang Y., Shi S., Zhao M., Chai X., Tu P. (2013). Coreosides A–D, *C*14-Polyacetylene Glycosides from the Capitula of *Coreopsis tinctoria* and Its Anti-Inflammatory Activity against COX-2. Fitoterapia.

[30] Du D., Jin T., Xing Z.-H., Hu L.-Q., Long D., Li S.-F., Gong M. (2016). One New Linear C14 Polyacetylene Glucoside with Antiadipogenic Activities on 3T3-L1 Cells from the Capitula of *Coreopsis tinctoria*. J. Asian Nat. Prod. Res..

[31] Magalhães A.F., Vieira D.M., Magalhães E.G., Shepherd G.J. (1988). C14 Polyacetylenes from Brazilian Lobelioideae. Phytochemistry.

[32] Kurimoto S., Okasaka M., Kashiwada Y., Kodzhimatov O.K., Takaishi Y. (2010). A C14-Polyacetylenic Glucoside with an α-Pyrone Moiety and Four C10-Polyacetylenic Glucosides from *Mediasia macrophylla*. Phytochemistry.

[33] Ngahang Kamte S.L., Ranjbarian F., Cianfaglione K., Sut S., Dall’Acqua S., Bruno M., Afshar F.H., Iannarelli R., Benelli G., Cappellacci L. (2018). Identification of Highly Effective Antitrypanosomal Compounds in Essential Oils from the Apiaceae Family. Ecotoxicol. Environ. Saf..

[34] Pavela R., Maggi F., Cianfaglione K., Bruno M., Benelli G. (2018). Larvicidal Activity of Essential Oils of Five Apiaceae Taxa and Some of Their Main Constituents Against *Culex quinquefasciatus*. Chem. Biodivers..

[35] Delazar A., Yari S.M., Chaparzadeh N., Asnaashari S., Nahar L., Delazar N., Sarker S.D. (2015). Chemical Composition, Free-Radical-Scavenging and Insecticidal Properties, and General Toxicity of Volatile Oils Isolated from Various Parts of *Echinophora orientalis*. J. Essent. Oil Bear. Plants.

[36] Thiviya P., Gunawardena N., Gamage A., Madhujith T., Merah O. (2022). Apiaceae Family as a Valuable Source of Biocidal Components and Their Potential Uses in Agriculture. Horticulturae.

[37] Ahmad V.U., Jassbi A.R., Pannahi M.S.C. (1999). Analysis of the Essential Oil of *Echinophora sibthorpiana* Guss. by Means of GC, GC/MS and 13C-NMR Techniques. J. Essent. Oil Res..

[38] Şanlı A., Karadogan T., Tosun B., Tonguç M., Erbaş S. (2016). Growth Stage and Drying Methods Affect Essential Oil Content and Composition of Pickling Herb (*Echinophora tenuifolia* subsp. sibthorpiana Tutin). SDÜ Fen Bilim. Enstitüsü Derg..

[39] Gokbulut I., Bilenler T., Karabulut I. (2013). Determination of Chemical Composition, Total Phenolic, Antimicrobial, and Antioxidant Activities of *Echinophora tenuifolia* Essential Oil. Int. J. Food Prop..

[40] Özcan M., Akgül A. (2003). Essential Oil Composition of Turkish Pickling Herb (*Echinophora tenuifolia* L. subsp. sibthorpiana (Guss.) Tutin). Acta Bot. Hung..

[41] Telci I., Hisil Y. (2008). Essential Oil Composition of the Spice Plant *Echinophora tenuifolia* L. Subsp. sibthorpiana Tutin from Turkey. Chem. Nat. Compd..

[42] Chalchat J.-C., Ozcan M., Dağdelen A., Akgul A. (2007). Variability of Essential Oil Composition of *Echinophora tenuifolia* subsp. sibthorpiana Tutin by Harvest Location and Year and Oil Storage. Chem. Nat. Compd..

[43] Chalchat J., Özcan M., Figueredo G., Chalard P. (2011). The Effect of Harvest Years on Chemical Composition of Essential Oil of Pickling Herb (*Echinophora tenuifolia* subsp. sibthorpiana) Leaves Used as Medicinal Plant. Acta Bot. Hung..

[44] Sanli A., Ok F.Z. (2023). Chemical Composition and Antimicrobial Activity against Phytopathogenic Fungi of Essential Oils Obtained from *Echinophora tenuifolia* subsp. *sibthorpiana* Grown in Wild and Cultivated Conditions in Turkey. Molecules.

[45] Papanikolaou N.E., Kavallieratos N.G., Iliopoulos V., Evergetis E., Skourti A., Nika E.P., Haroutounian S.A. (2022). Essential Oil Coating: Mediterranean Culinary Plants as Grain Protectants against Larvae and Adults of *Tribolium castaneum* and *Trogoderma granarium*. Insects.

[46] Evergetis E., Michaelakis A., Haroutounian S.A. (2013). Exploitation of Apiaceae Family Essential Oils as Potent Biopesticides and Rich Source of Phellandrenes. Ind. Crops Prod..

[47] Glamočlija J.M., Soković M.D., Šiljegović J.D., Ristić M.S., Ćirić A.D., Grubišić D.V. (2011). Chemical Composition and Antimicrobial Activity of *Echinophora spinosa* L. (Apiaceae) Essential Oil. Rec. Nat. Prod..

[48] Fayyaz N., Mohamadi Sani A., Najaf Najafi M. (2015). Antimicrobial Activity and Composition of Essential Oil from *Echinophora platyloba*. J. Essent. Oil Bear. Plants.

[49] Ghasemi Pirbalouti A., Gholipour Z. (2016). Chemical Composition, Antimicrobial and Antioxidant Activities of Essential Oil from *Echinophora cinerea* Harvested at Two Phenological Stages. J. Essent. Oil Res..

[50] Sajjadi S.E., Ghannadi A. (2002). Composition of the Essential Oil of *Echinophora cinerea* (Boiss.) Hedge et Lamond. J. Essent. Oil Res..

[51] Jahantab E., Morshedloo M.R., Karimian V., Sharafatmandrad M. (2022). Essential Oil Variability in *Echinophora cinerea* Boiss. Wild Populations: A Narrow- Endemic and Vulnerable Species in Iran. J. Essent. Oil Res..

[52] Ahmadi L., Mirza M., Khorram M.T. (2001). Essential Oil of *Echlnophora cinerea* (Boiss.) Hedge and Lamond from Iran. J. Essent. Oil Res..

[53] Hashemi P., Abolghasemi M.M., Ghiasvand A.R., Ahmadi S., Hassanvand H., Yarahmadi A. (2009). A Comparative Study of Hydrodistillation and Hydrodistillation–Solvent Microextraction Methods for Identification of Volatile Components of *Echinophora cinerea*. Chromatographia.

[54] Sodeifian G., Sajadian S.A. (2017). Investigation of Essential Oil Extraction and Antioxidant Activity of *Echinophora platyloba* DC. Using Supercritical Carbon Dioxide. J. Supercrit. Fluids.

[55] Gholivand M.B., Rahimi-Nasrabadi M., Mehraban E., Niasari M., Batooli H. (2011). Determination of the Chemical Composition and in Vitro Antioxidant Activities of Essential Oil and Methanol Extracts of *Echinophora platyloba* DC. Nat. Prod. Res..

[56] Saei-Dehkordi S.S., Fallah A.A., Saei-Dehkordi S.S., Kousha S. (2012). Chemical Composition and Antioxidative Activity of *Echinophora platyloba* DC. Essential Oil, and Its Interaction with Natural Antimicrobials against Food-Borne Pathogens and Spoilage Organisms. J. Food Sci..

[57] Hashemi M., Ehsani A., Hosseini Jazani N., Aliakbarlu J., Mahmoudi R. (2013). Chemical Composition and In Vitro Antibacterial Activity of Essential Oil and Methanol Extract of *Echinophora platyloba* D.C against Some of Food-Borne Pathogenic Bacteria. Vet. Res. Forum.

[58] Moghaddam M., Taheri P., Pirbalouti A.G., Mehdizadeh L. (2015). Chemical Composition and Antifungal Activity of Essential Oil from the Seed of *Echinophora platyloba* DC. against Phytopathogens Fungi by Two Different Screening Methods. LWT-Food Sci. Technol..

[59] Sharifian I., Darvishzadeh A. (2015). Chemical Composition and Insecticidal Efficacy of Essential Oil of *Echinophora platiloba* DC (Apiaceae) from Zagros Foothills, Iran. Arthropods.

[60] Baser K.H.C., Özek T., Demirçakmak B., Biçakçi A., Malyer H. (1996). Essential Oil of *Echinophora chrysantha* Freyn et Sint. J. Essent. Oil Res..

[61] Baser K.H.C., Kürkcüoglu M., Malyer H., Bicakci A. (1998). Essential Oils of Six *Echinophora* Species from Turkey. J. Essent. Oil Res..

[62] Akşit Z., Fırat M.Ç., Akşit H., Bayar Y., Alkan M., Şimşek S. (2024). Chemical Composition, Antifungal, Antibacterial, and Insecticidal Activity of *Echinophora chrysantha* Essential Oil. J. Essent. Oil Bear. Plants.

[63] Demirci B., Kiyan T., Koparal A., Kaya M., Demirci F., Baser K. (2010). The In Vivo and In Vitro Angiogenic Evaluation of the Essential Oil of *Echinophora tournefortii*. Planta Med..

[64] Baser K.H.C., Biçakçi A., Malyer H. (2000). Composition of the Essential Oil of *Echinophora lamondiana* B. Yildiz et Z.Bahçecioglu. J. Essent. Oil Res..

[65] Ali A., Tabanca N., Ozek G., Ozek T., Aytac Z., Bernier U.R., Agramonte N.M., Baser K.H.C., Khan I.A. (2015). Essential Oils of *Echinophora lamondiana* (Apiales: Umbelliferae): A Relationship Between Chemical Profile and Biting Deterrence and Larvicidal Activity Against Mosquitoes (Diptera: Culicidae). J. Med. Entomol..

[66] Avijgan M., Mahboubi M. (2015). *Echinophora platyloba* DC. as a New Natural Antifungal Agent. Asian Pac. J. Trop. Dis..

[67] Ghafoor K., Al-Juhaimi F., Özcan M.M., Babiker E.E., Ahmed I.A.M., Alsawmahi O.N. (2021). Bioactive Compounds, Antioxidant Activity and Sensory Properties of Tarhana, a Traditional Fermented Food, Enriched with Pickling Herb (*Echinophora tenuifolia* L.). Int. J. Food Sci. Technol..

[68] Ghasemi Pirbalouti A., Malekpoor F., Enteshari S., Yousefi M., Momtaz H., Hamedi B. (2010). Antibacterial Activity of Some Folklore Medicinal Plants Used by Bakhtiari Tribal in Southwest Iran. Int. J. Biol..

[69] Tahvilian R., Shahriari S., Faramarzi A., Komasi A. (2014). Ethno-Pharmaceutical Formulations in Kurdish Ethno-Medicine. Iran. J. Pharm. Res..

[70] Abbasi N., Rafieian-Kopaei M., Karami N., Ghanadi K. (2019). An Ethnobotanical Study of Hepatoprotective Herbs from Shahrekord, Chaharmahal and Bakhtiari Province, Southwest of Iran. Egypt. J. Vet. Sci..

[71] Anbari K., Abbaszadeh S., Basati G. (2019). Medicinal Plants with Preventive and Therapeutic Effect on Diarrhoea: A Cross-Sectional Epidemiologic and Ethnobotanical Study in Traditional Therapists of Shahrekord, South-West of Iran. Plant Sci. Today.

[72] Moghanloo L., Ghahremaninejad F., Vafadar M. (2019). Ethnobotanical Study of Medicinal Plants in the Central District of the Zanjan County, Zanjan Province, Iran. J. Med. Herbs.

[73] Mosaddegh M., Esmaeili S., Hassanpour A., Malekmohammadi M., Naghibi F. (2016). Ethnobotanical Study in the Highland of Alvand and Tuyserkan, Iran. Res. J. Pharmacogn..

[74] Kheirollahi A.R., Mahmoodnia L., Khodadustan E., Kazemeini H., Hasanvand A., Hatamikia M. (2019). Medicinal Plants for Kidney Pain: An Ethnobotanical Study on Shahrekord City, West of Iran. Plant Sci. Today.

[75] Kivanç M. (1988). Antimicrobial activity of “Çörtük” (*Echinophora sibthorpiana* Guss.) spice, its essential oil and methyl-eugenol (Short communication). Food/Nahr..

[76] Kargıoğlu M., Cenkci S., Serteser A., Evliyaoğlu N., Konuk M., Kök M.Ş., Bağcı Y. (2008). An Ethnobotanical Survey of Inner-West Anatolia, Turkey. Hum. Ecol..

[77] Yeşil Y., Çelik M., Yılmaz B. (2019). Wild Edible Plants in Yeşilli (Mardin-Turkey), a Multicultural Area. J. Ethnobiol. Ethnomed..

[78] Ahmet Doğan G.B., Doğan A., Bulut G., Tuzlacı E., Şenkardeş İ. (2015). A Review of Edible Plants on the Turkish Apiaceae Species. J. Fac. Pharm. Istanb. Univ..

[79] Georgala A. (2012). The Nutritional Value of Two Fermented Milk/Cereal Foods Named ‘Greek Trahanas’ and ‘Turkish Tarhana’: A Review. J. Nutr. Disord. Ther..

[80] Akbulut S., Karakose M., Özkan Z.C. (2019). Traditional Uses of Some Wild Plants in Kale and Acıpayam Provinces in Denizli. Kastamonu Univ. J. For. Fac..

[81] Ozdemir S., Gocmen D., Yildirim Kumral A. (2007). A Traditional Turkish Fermented Cereal Food: Tarhana. Food Rev. Int..

[82] Deghirmencioghlu N., Gocmen D., Dağdelen A., Dağdelen F. (2005). Influence of Tarhana Herb (*Echinophora sibthorpiana*) on Fermentation of Tarhana, Turkish Traditional Fermented Food. Food Technol. Biotechnol..

[83] Cakilcioglu U., Turkoglu I. (2010). An Ethnobotanical Survey of Medicinal Plants in Sivrice (Elazığ-Turkey). J. Ethnopharmacol..

[84] Erarslan Z.B., Çolak R., Kültür Ş. (2021). The Preliminary Ethnobotanical Survey of Medicinal Plants in Develi (Kayseri/Turkey). IUJP.

[85] Ünsal Ç., Vural H., Sariyar G., Özbek Çelik B., Ötük G. (2010). Traditional Medicine in Bïlecïk Province (Turkey) and Antimicrobial Activities of Selected Species. Turk. J. Pharm. Sci..

[86] Bulut G., Haznedaroğlu M.Z., Doğan A., Koyu H., Tuzlacı E. (2017). An Ethnobotanical Study of Medicinal Plants in Acipayam (Denizli-Turkey). J. Herb. Med..

[87] Yeşilada E., Honda G., Sezik E., Tabata M., Goto K., Ikeshiro Y. (1993). Folk Medicine in the Mediterranean Subdivision. J. Ethnopharmacol..

[88] Fakir H., Korkmaz M., Güller B. (2009). Medicinal Plant Diversity of Western Mediterrenean Region in Turkey. J. Appl. Biol. Sci..

[89] Salas-Oropeza J., Jimenez-Estrada M., Perez-Torres A., Castell-Rodriguez A.E., Becerril-Millan R., Rodriguez-Monroy M.A., Jarquin-Yañez K., Canales-Martinez M.M. (2021). Wound Healing Activity of α-Pinene and α-Phellandrene. Molecules.

[90] Chakarov D., Hadzhieva E., Kalchev Y., Hadzhiev D. (2024). Aerobic Microbiological Spectrum and Antibiotic Resistance in Children Operated for Anorectal Abscesses. J. Clin. Med..

[91] Khalid U., Uchikov P., Hristov B., Kraev K., Koleva-Ivanova M., Kraeva M., Batashki A., Taneva D., Doykov M., Uchikov A. (2024). Surgical Innovations in Tracheal Reconstruction: A Review on Synthetic Material Fabrication. Medicina.

[92] Costa M.F., Durço A.O., Rabelo T.K., Barreto R.d.S.S., Guimarães A.G. (2019). Effects of Carvacrol, Thymol and Essential Oils Containing Such Monoterpenes on Wound Healing: A Systematic Review. J. Pharm. Pharmacol..

[93] Chaleshtori R., Rafieian-kopaei M., Mortezaei S., Sharafati-Chaleshtori A., Amini E. (2012). Antioxidant and Antibacterial Activity of the Extracts of *Echinophora platyloba* D.C. Afr. J. Pharm. Pharmacol..

[94] Avijgan M., Mohaddese M., Mahdi D., Mahdi S., Sanaz S., Kassaiyan N. (2010). Overview on *Echinophora platyloba*, a Synergistic Anti-Fungal Agent Candidate. J. Yeast Fungal Res..

[95] Avijgan M., Hafizi M., Mehdi S., Nilforoushzadeh M.A. (2006). Antifungal Effect of *Echinophora platyloba*’ s Extract against Candida Albicans. Iran. J. Pharm. Res..

[96] Sepehri Z., Javadian F., Khammari D., Hassanshahian M. (2016). Antifungal Effects of the Aqueous and Ethanolic Leaf Extracts of *Echinophora platyloba* and *Rosmarinus officinalis*. Curr. Med. Mycol..

[97] Khajeh E., Hosseini Shokouh S.J., Rajabibazl M., Roudbary M., Rafiei S., Aslani P., Farahnejad Z. (2016). Antifungal Effect of *Echinophora platyloba* on Expression of CDR1 and CDR2 Genes in Fluconazole-Resistant Candida Albicans. Br. J. Biomed. Sci..

[98] Avijgan M., Mahboubi M., Moheb Nasab M., Ahmadi Nia E., Yousefi H. (2014). Synergistic Activity between *Echinophora platyloba* DC Ethanolic Extract and Azole Drugs against Clinical Isolates of Candida Albicans from Women Suffering Chronic Recurrent Vaginitis. J. Mycol. Med..

[99] Avijgan M., Mirzadeh F., Nia E. (2012). The Comparative Study of Anti-Fungal Effect of Pharmaceutical Products Containing Hydroalcoholic Extract of *Echinophora platyloba* DC and Fluconazole in Women with Chronic Recurrent Vaginitis Caused by *Candida albicans*. Res. J. Med. Sci..

[100] Rashidi F., Ganbalani G.N. (2018). Toxicity and Sublethal Effects of Selected Insecticides on Life Parameters of *Encarsia formosa* (Hymenoptera: Aphelinidae), a Parasitoid of *Trialeurodes vaporariorum* (Hemiptera: Aleyrodidae). J. Entomol. Sci..

[101] Shahneh F.Z., Valiyari S., Azadmehr A., Hajiaghaee R., Yaripour S., Bandehagh A., Baradaran B. (2013). Inhibition of Growth and Induction of Apoptosis in Fibrosarcoma Cell Lines by *Echinophora platyloba* DC: In Vitro Analysis. Adv. Pharmacol. Sci..

[102] Birjandian E., Motamed N., Yassa N. (2018). Crude Methanol Extract of *Echinophora platyloba* Induces Apoptosis and Cell Cycle Arrest at S-Phase in Human Breast Cancer Cells. Iran. J. Pharm. Res..

[103] Entezari M., Dabaghian F.H., Hashemi M. (2014). The Comparison of Antimutagenicity and Anticancer Activities of *Echinophora platyloba* DC on Acute Promyelocytic Leukemia Cancer Cells. J. Cancer Res. Ther..

[104] Amirghofran Z., Bahmani M., Azadmehr A., Javidnia K. (2006). Anticancer Effects of Various Iranian Native Medicinal Plants on Human Tumor Cell Lines. Neoplasma.

[105] Asgari Nematian M., Mohammadi S. (2016). The Analgesic Effect of *Echinophora platyloba* Hydroalcoholic Extract in Male Rats. J. Babol Univ. Med. Sci..

[106] Valatabar P., Khezri S., Barzegari A. (2023). Effects of hydro-ethanolic extract of *Echinophora platyloba* L. (Apiaceae) on the expression and acquisition of morphine-induced place conditioning in female mice. Trends Phytochem. Res..

[107] Delaram M., Sadeghiyan Z. (2010). The Effect of Echinophora-Platyloba Extract on Primary of Dysmenorrhea. J. Arak Univ. Med. Sci..

[108] Mirabi P., Alamolhoda S.H., Esmaeilzadeh S., Mojab F. (2014). Effect of Medicinal Herbs on Primary Dysmenorrhoea- a Systematic Review. Iran. J. Pharm. Res..

[109] Delaram M. (2014). Treatment of Moderate to Severe of Premenstrual Syndrome with *Echinophora platyloba*. Zahedan J. Res. Med. Sci..

[110] Mansour S., Ali Z., Saeed C.-A. (2016). A Study on the Effects of the Alcoholic Extract of the Aerial Parts of *Echinophora platyloba* on the Activity of Pituitary-Gonadal Axis in Hypercholesterolemic Rats. J. Appl. Pharm. Sci..

[111] Heidarian E., Saffari J., Jafari-Dehkordi E. (2014). Hepatoprotective Action of *Echinophora platyloba* DC Leaves Against Acute Toxicity of Acetaminophen in Rats. J. Diet. Suppl..

[112] Amirghofran Z., Bahmani M., Azadmehr A., Javidnia K., Miri R. (2009). Immunomodulatory Activities of Various Medicinal Plant Extracts: Effects on Human Lymphocytes Apoptosis. Immunol. Investig..

[113] Fraternale D., Ricci D., Calcabrini C., Guescini M., Martinelli C., Sestili P. (2013). Cytotoxic Activity of Essential Oils of Aerial Parts and Ripe Fruits of *Echinophora spinosa* (Apiaceae). Nat. Prod. Commun..

